# Supplementary material for: A Humanized Yeast Phenomic Model of Deoxycytidine Kinase to Predict Genetic Buffering of Nucleoside Analog Cytotoxicity
Source: Genes (Basel). 2019 Sep 30;10(10):770. doi: 10.3390/genes10100770 (PMC6826991; doi:10.3390/genes10100770)
Supplement: Supplementary file 1 [file genes-10-00770-s001.zip › Additional_Files/Additional_File7_GOTermHeatmaps.zip!/Additional_File7_GOTermHeatmaps/nuclear_chromosome.pdf]

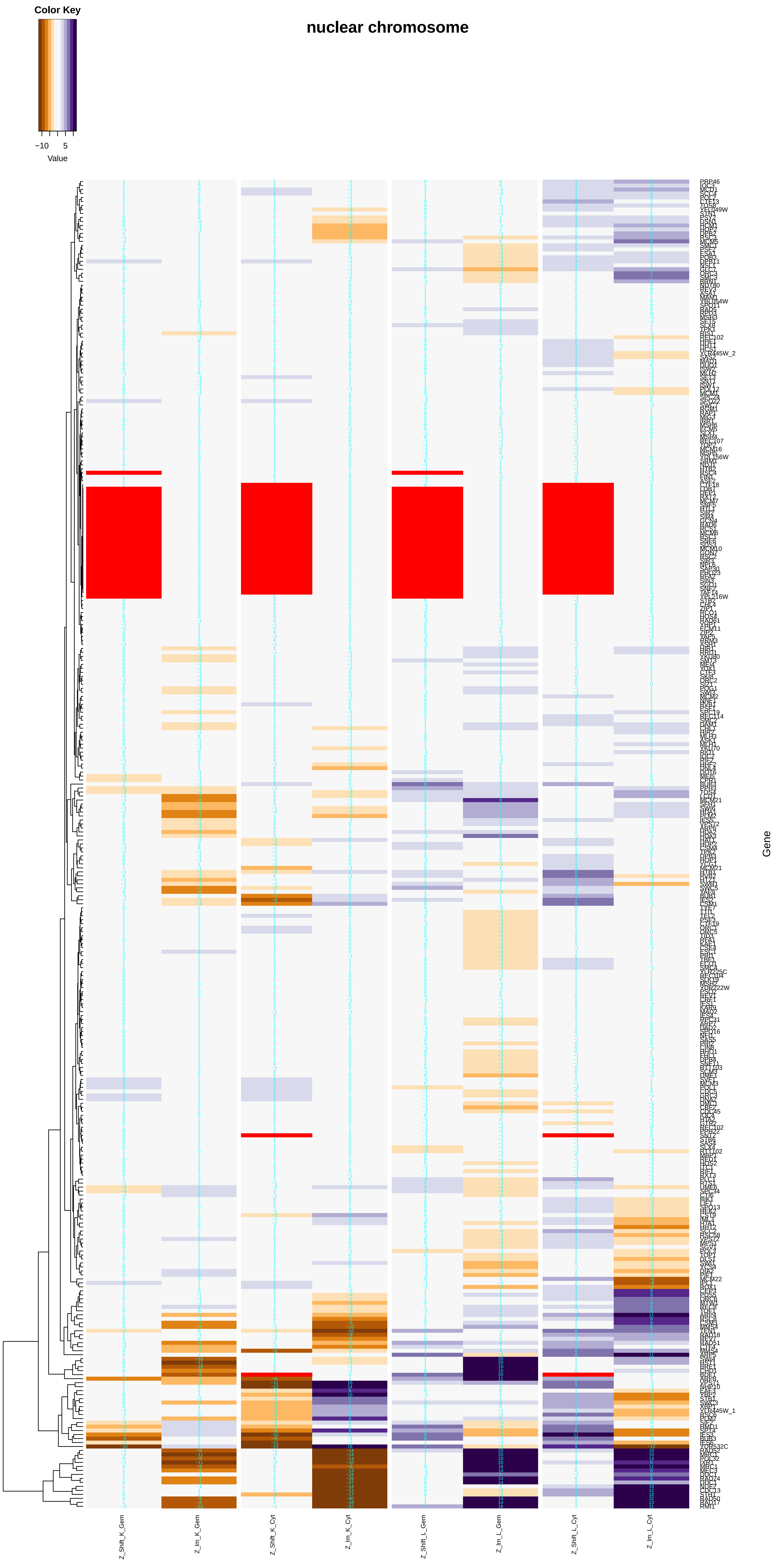
nuclear chromosome
Color Key
−10
5
Value
Gene
Z_Shift_K_Gem
Z_Im_K_Gem
Z_Shift_K_Cyt
Z_Im_K_Cyt
Z_Shift_L_Gem
Z_Im_L_Gem
Z_Shift_L_Cyt
Z_Im_L_Cyt

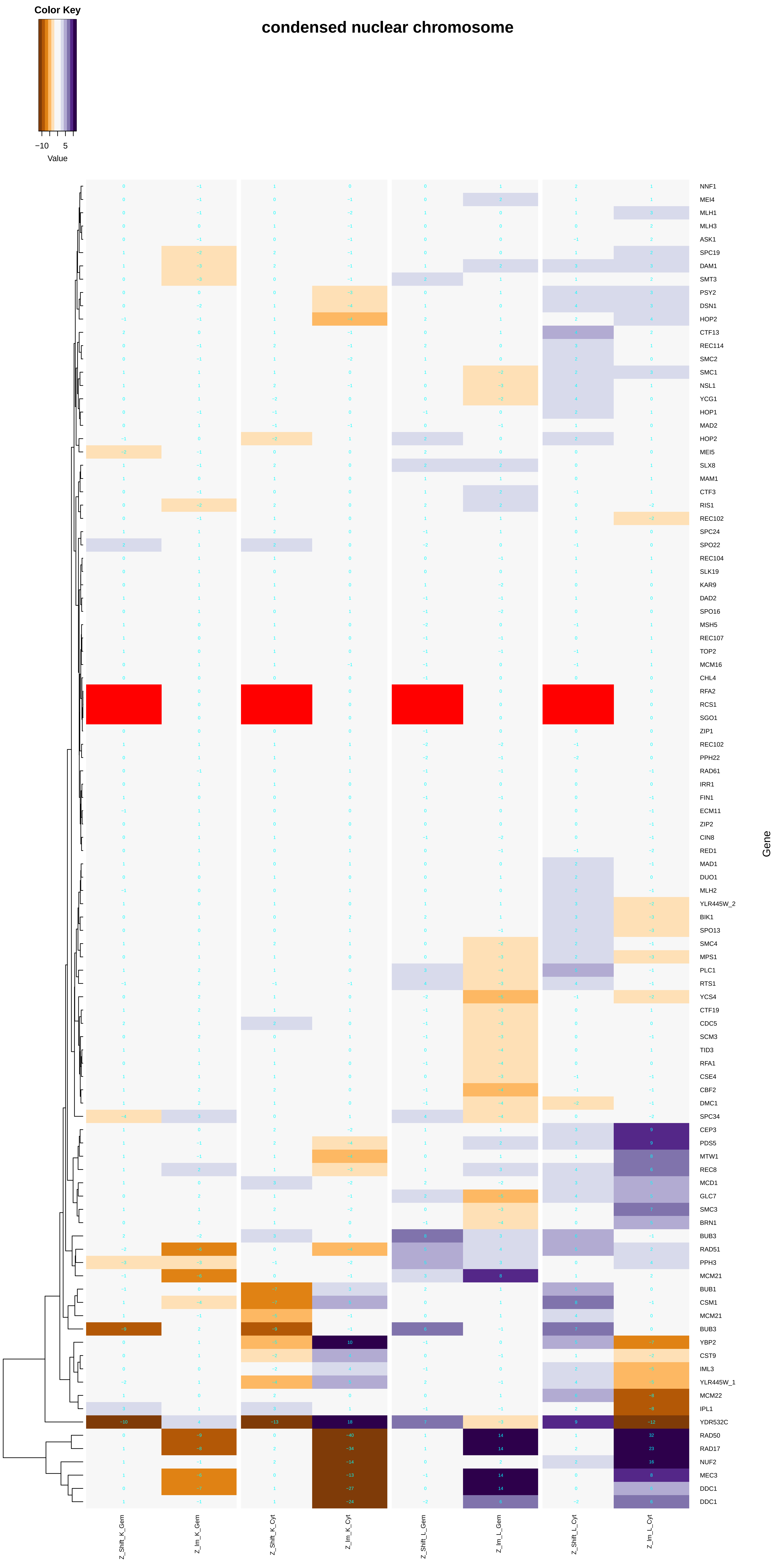
Color Key
−10
5
Value
condensed nuclear chromosome
NNF1
MEI4
MLH1
MLH3
ASK1
SPC19
DAM1
SMT3
PSY2
DSN1
HOP2
CTF13
REC114
SMC2
SMC1
NSL1
YCG1
HOP1
MAD2
HOP2
MEI5
SLX8
MAM1
CTF3
RIS1
REC102
SPC24
SPO22
REC104
SLK19
KAR9
DAD2
SPO16
MSH5
REC107
TOP2
MCM16
CHL4
RFA2
RCS1
SGO1
ZIP1
REC102
PPH22
RAD61
IRR1
FIN1
ECM11
ZIP2
CIN8
RED1
MAD1
DUO1
MLH2
YLR445W_2
BIK1
SPO13
SMC4
MPS1
PLC1
RTS1
YCS4
CTF19
CDC5
SCM3
TID3
RFA1
CSE4
CBF2
DMC1
SPC34
CEP3
PDS5
MTW1
REC8
MCD1
GLC7
SMC3
BRN1
BUB3
RAD51
PPH3
MCM21
BUB1
CSM1
MCM21
BUB3
YBP2
CST9
IML3
YLR445W_1
MCM22
IPL1
YDR532C
RAD50
RAD17
NUF2
MEC3
DDC1
DDC1
Gene
Z_Shift_K_Gem
Z_Im_K_Gem
Z_Shift_K_Cyt
Z_Im_K_Cyt
Z_Shift_L_Gem
Z_Im_L_Gem
Z_Shift_L_Cyt
Z_Im_L_Cyt
